# Theoretical Prediction of Electrical Conductivity Percolation of Poly(lactic acid)—Carbon Nanotube Composites in DC and RF Regime

**DOI:** 10.3390/ma16155356

**Published:** 2023-07-30

**Authors:** Freddys R. Beltrán, Hammouche Aksas, Lakhdar Sidi Salah, Yann Danlée, Isabelle Huynen

**Affiliations:** 1 Departamento Ingeniería Química Industrial y Medio Ambiente, E.T.S.I. Industriales, Universidad Politécnica de Madrid, 28006 Madrid, Spain; f.beltran@upm.es; 2 Research Group “Polímeros, Caracterización y Aplicaciones (POLCA)”, Universidad Politécnica de Madrid, 28006 Madrid, Spain; 3 Research Unit Materials, Processes and Environment (URMPE), Faculty of Technology, M’Hamed Bougara University, Boumerdes 35000, Algeria; h.aksas@univ-boumerdes.dz; 4 Institute of Information and Communication Technologies, Electronics and Applied Mathematics (ICTEAM), Université Catholique de Louvain, Place du Levant 3, 1348 Louvain-la-Neuve, Belgium; yann.danlee@uclouvain.be

**Keywords:** percolation, polylactic acid, carbon nanotube, composite, modelling, electrical conductivity, microwave

## Abstract

Polymer composites based on polylactic acid (PLA) reinforced with 0.25–5 wt.% of carbon nanotubes (CNTs) were synthesized by melt blending. The static (DC) and microwave (RF) electrical conductivity have been investigated on the PLA–CNT composites. The electrical percolation threshold has been theoretically determined using classical models of percolation in order to predict the conductivity of the different nanocomposites. Through the fitting process, it has been found that the percolation threshold is obtained at 1 wt.% of CNTs in the DC regime and reached below 0.25 wt.% of CNTs in the microwave regime. Among the Mamunya, McLachlan, or GEM models, the McCullough model remarkably fits the experimental DC and RF electrical conductivities. The obtained results are correlated to the electrical properties of a range of CNT-based composites, corresponding to the percolation threshold required for a three-dimensional network of CNTs into the polymer matrix.

## 1. Introduction

Carbon nanotubes (CNTs) blended into polymer nanocomposites are very relevant thanks to their tunable conductivity and advantageous properties, depending on the number of CNTs and the nature of the polymer matrix [1]. CNTs exhibit significant electrical conductivity; they can reach $10^{6}$ S/m. On the other hand, polymeric matrices are insulating as their conductivity ranges between $10^{-16}$ and $10^{-12}$ S/m [2]. The addition of a very low number of CNTs to the polymer significantly increases the effective conductivity of the resulting nanocomposite [3,4]. The application of conductive polymer composites (CPCs) as potential innovative micro-devices is increasing, especially in electronics for sensors and actuators as well as several other applications [5,6,7,8]. The field of radar-absorbing materials (RAMs) mainly uses carbon nanotube composites since they offer a high absorption index with low thickness, flexibility, and good mechanical properties [9,10]. Despite the nature of these materials, their architecture also allows one to improve the efficiency of electromagnetic interference (EMI) shielding [11,12].

The percolation threshold is referred to as a critical filler concentration that induces high changes in conductivity, and the adjacent nanoparticles form a continuous conductive pathway within the nanocomposite [13,14]. The electrical characterization of the effective conductivity at different filler concentrations allows one to determine the percolation threshold [15]. The scientific literature commonly relates the percolation threshold to the size of the one-dimensional nanofiller material [16,17]. Besides the aspect ratio of the nanofillers, their nature, and their structure, the quality of the dispersion, driven by the interphase properties, influences the percolation level [15,18]. The implementation process can lead to to an anisotropic effective conductivity. Mechanical stretching, squeezing, or any method of nanocharge alignment causes one- or two-dimensional anisotropy, which opens the way to interesting applications such as the radar polarizer [19,20].

The behavior of the effective complex permittivity of conductive polymer composites (CPCs) is expected to be well predicted by the percolation model in the DC regime [21] as well as at low and high microwave frequencies [22,23]. Several theoretical models have been applied to predict the conductivity of the CPCs in order to check their validity and limitations [24]. For instance, in our previous work, different models based on mathematical expressions were used to describe the RF electrical conductivity of polycarbonate composites [25]. Since there is no standard model that fits all formulations, a set of theoretical models is applied to accurately predict the conductivity for the different CPCs [26]. The verification of such existing theoretical models is necessary to know about their applicability and limitations [27,28].

This study focuses on understanding the electrical and dielectric properties of PLA–CNT composites within the scope of the percolation theory, in order to design better composites including multi-walled carbon nanotubes (MWCNTs). One standout feature of our research is the meticulous examination of both the static and RF electrical conductivity of the PLA–CNT composites. This level of thorough investigation, which, to our knowledge, has not been previously undertaken, paves the way for a deeper understanding of these composites’ potential uses, especially in the ESD [29] or EMI shielding [30] fields, where electrical conductivity is vital. The theoretical determination of the percolation threshold using classical percolation models fulfils the current knowledge regarding the electrical conductivity of nanocomposites. The successful application of the models to fit experimental DC and RF electrical conductivity results opens an avenue to broader applications in future composite materials research, enhancing the consistency of research outcomes and predictions. This study reports the current experimental results in the DC and RF regimes and provides a discussion of the percolation parameters supported by various models of the electrical conductivity.

## 2. Materials and Methods

### 2.1. Materials and Compounds

The material used in this study was a commercially available PLA, concretely the Ingeo™ Biopolymer 2003D, purchased to NatureWorks (Savage, USA). It had a melt mass-flow rate of 6 g/10 min (2.16 kg at 210 °C). The conductive nanofillers used were multi-walled NC7000 carbon nanotubes obtained from NanoCyl SA (Sambreville, Belgium) using the catalytic chemical vapor deposition (CCVD) method. The density of PLA was 1.24 g/cm^3^ (source: https://www.natureworksllc.com/~/media/Technical_Resources/Technical_Data_Sheets/TechnicalDataSheet_2003D_FFP-FSW_pdf.pdf accessed on 20 July 2023) and the one of pure CNTs was 1.75 g/cm^3^ (source: https://www.nanocyl.com/product/nc7000/ accessed on 20 July 2023). Different weight or equivalent volume concentrations of CNTs were added, as detailed in Table 1.

### 2.2. Preparation of the Materials

The PLA pellets and CNT nanoparticles were combined by melt extrusion in a Rondol Microlab twin-screw microcompounder (L/D = 20) operating at 60 rpm. The temperature profile, starting from the hopper and moving towards the die, was 125—165—190—190—180 °C. The resulting material was subsequently transformed into 200 µm-thick films by hot-pressing using an IQAP-LAP hot plate press at a temperature of 190 °C.

### 2.3. Characterization Methods

The in-plane DC conductivity was measured by a 4-probe method using a Keithley K2400 or K6430 equipped with a SP4 Four Point Probe Head (Lucas Labs). The quite high resistance of the composite at the DC regime prevented one from performing systematic measurements of I-V curves because it was close to the limit of the instrument. The 4-probe was thus realized at one voltage, which prevented I-V curves. The probes were arranged linearly in a straight line at equidistance of 1 mm from each other. Assuming that the electron propagation depth is smaller than the gap between probes, which is totally satisfied since the thickness itself of the samples is often thinner than this gap, we can use the formula for effective conductivity:

(1) $$\sigma_{DC}=\frac{\ln(2)}{\pi }\frac{U}{I}$$

The in-plane microwave (RF) conductivity for different volume fractions of CNT is obtained from electromagnetic characterization using the Anritsu M54644B vector network analyzer (VNA) in waveguide configuration over the Ka band. The calibration was made by LRL/LRM method, and the IF bandwidth was set at 300 Hz (for additional information about the calibration mode used with the VNA, see https://dl.cdn-anritsu.com/en-us/test-measurement/files/Application-Notes/Application-Note/11410-00492C.pdf accessed on 20 July 2023).

The instrument provides S-parameters that are converted to physical parameters following the formalism described in ref. [31] and consolidated by homemade algorithms generalized to magnetic materials [32]. The main concern in this work is the extracted RF conductivity $\sigma_{RF}$ defined by complex permittivity:

(2) $$\varepsilon_{rRF}=\varepsilon_{rRF}^{\prime }-\varepsilon_{rRF}^{\prime \prime }=\varepsilon_{rRF}^{\prime }-\frac{j.\sigma_{RF}}{\omega .\varepsilon_{0}}$$

where $\varepsilon_{rRF}^{\prime }$ is the RF dielectric constant, ω is the pulsation, and $\varepsilon_{0}$ is the vacuum permittivity.

## 3. Theoretical Models

The percolation threshold is determined by plotting the conductivity as a function of the CNT loading. The percolation is observed when an electrical percolation path is formed between the nanofillers. This is realized when a well dispersed nanofiller loading in a dielectric matrix imparts an increase in effective conductivity. Moreover, high frequency causes tunneling adjacent CNTs (comparable to capacitors) to form a percolation path [33,34,35]. The empirical data fit is basically used in DC; nevertheless, the observed behavior is similar, making the same formalism applicable in the microwave range [12,14]:

(3) $$\sigma =k{\left(\varphi -\varphi_{c}\right)}^{\mu }\;for\;\varphi >\varphi_{c}$$

where *k* and μ are fitting constants, φ is the volume fraction of reinforcement, and $\varphi_{c}$ is the percolation threshold [14]. μ accurately represents the dimensionality of the composite, and in some studies *k* represents the conductivity of the matrix saturated by the nanofiller, but this interpretation is not generalized for all the nanocomposite systems. One considers μ as the host conductivity when the nanofiller and the matrix do have not a large difference in conductivity. In our case, the nanofiller is ultra-conductive and the matrix is insulting. Equation (3) can be rewritten by taking the logarithm of both sides:

(4) $$\log\sigma =\log k+\mu \log\left(\varphi -\varphi_{c}\right)$$

Mamunya et al. [36] have developed a model based on the evaluation of polymer–filler interaction on the conductivity beyond the percolation threshold. The model is expressed by Equation (5).

(5) $$\log\sigma_{c}=\log\sigma_{\varphi c}+\left(\log\sigma_{max}-\log\sigma_{\varphi c}\right){\left(\frac{\varphi -\varphi_{c}}{F-\varphi_{c}}\right)}^{k}$$

where $\sigma_{c}$, $\sigma_{\varphi ,c}$, and $\sigma_{max}$ are the electrical conductivity of the composite, of the composite at the percolation threshold, and of the composite at a maximum volume fraction of filler, respectively. *F* is associated with the maximum volume fraction of the CNT (packing factor) loading used in our study and is defined here below:

(6) $$F=\frac{5}{\frac{75}{10+AR}+AR}$$

where *AR* is the aspect ratio.

It should be noted that the *k* coefficients in the percolation law and in the Mamunya model are different since these two models are mathematically different. Consequently, we cannot presume that we will get the same value of *k*. According to the percolation theory, *k* represents the three-dimensional network, while it is a parameter giving an idea about interfacial interaction between the nanofiller and the matrix for the Mamunya model.

The transport phenomena for predicting the conductivity is established by the Bueche equation [37,38]. McCullough has added components to this model in order to enhance the accuracy of the targeted property. The final formula for accurately predicting the effective conductivity of a composite according to the McCullough model is as follows:

(7) $$\sigma_{c}=\sigma_{p}\varphi_{p}+\sigma_{f}\varphi_{f}-\left(\frac{\lambda \varphi_{p}\varphi_{f}{\left(\sigma_{f}-\sigma_{p}\right)}^{2}}{(N_{f}\sigma_{f}+N_{p}\sigma_{p})}\right)$$

where $\sigma_{c}$ is the conductivity of the composite, $\sigma_{f}$ is the conductivity of the filler, $\sigma_{p}$ is the conductivity of pure polymeric matrix, and $\varphi_{f}$ and $\varphi_{p}$ are the volume fraction of the filler and polymer, respectively. $N_{f}$ and $N_{p}$ are defined as:

(8) $$\begin{array}{c} N_{f}=\left(1-\lambda \right)\varphi_{f}+\varphi_{p}\lambda \end{array}$$

(9) $$\begin{array}{c} N_{p}=\left(1-\lambda \right)\varphi_{p}+\varphi_{f}\lambda \end{array}$$

where λ is a structure factor that indicates the extent of conducting chain and network formation; its value varies from 0 to 1 [39].

The generalized effective medium (GEM) model developed by McLachlan was used to predict the electrical conductivity of homogeneous binary systems where the electrical conductivity of composites is given by [40]:

(10) $$\left(1-\varphi \right)\frac{\sigma_{h}^{\frac{1}{t}}-\sigma^{\frac{1}{t}}}{\sigma_{h}^{\frac{1}{t}}+\frac{\varphi -\varphi {}_{c}}{\varphi_{c}}\sigma^{\frac{1}{t}}}+\varphi \frac{\sigma_{f}^{\frac{1}{t}}-\sigma^{\frac{1}{t}}}{\sigma_{f}^{\frac{1}{t}}+\frac{\varphi -\varphi {}_{c}}{\varphi_{c}}\sigma^{\frac{1}{t}}}=0$$

where φ and $\varphi_{c}$ are the volume filler fraction and the percolation threshold; $\sigma_{h}$ and $\sigma_{f}$ are, respectively, the conductivity of the matrix and the filler; and *t* is the dimensionality of the percolating system.

## 4. Results and Discussion

### 4.1. Prediction of the Percolation Threshold in DC Regime

The PLA is a semi-crystalline polymer; the evolution of crystallinity could influence the electrical conductivity since the amorphous phase and the repartition of crystalline structures could play an important role in the evolution of the insulator behavior of the polymer layer between the CNTs. However, our investigations have shown a variation of crystallinity from 1% to 2%, which is negligible for PLA. Those values are in the error range of the technique. The crystallinity is consequently not considered in the discussion.

**Figure 1:**
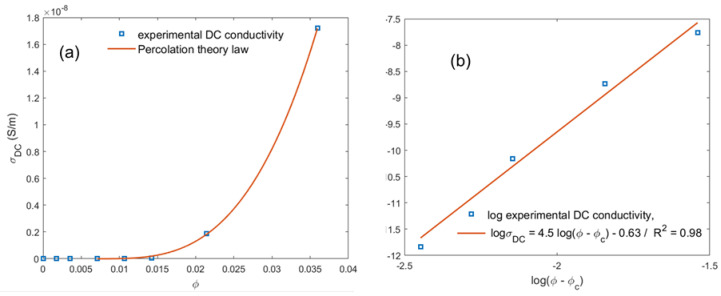
Electrical conductivity of PLA–CNT in function of vol.% of CNTs displayed in linear scale in (**a**); plot of $\log\sigma_{DC}$ in function of $\log\left(\varphi -\varphi_{c}\right)$ in (**b**).

The in-plane conductivity was measured for the PLA–CNT films described in Table 1. The variation of the static conductivity $\sigma_{DC}$ as a function of the filler volume loading φ is shown in Figure 1a. When φ<0.71 vol.% CNT, the PLA–CNT composites are the insulator and the conductivity is comparable to unmodified PLA. Practically, it was not possible to measure the resistivity of thin films along the plane because of the instrument limit, which is approximately $2.1\times 10^{-13}$ S/m. The results below $\varphi_{c}$ were consequently not precisely quantifiable. There were a few points of measurement (5 points per film), all showing a resistivity beyond the limits. The dominant resistivity of PLA might vary as the one reported by Bowen Yu et al. [41], but the phenomenon cannot be observed in our work. At φ>0.71 vol.% CNT, a sharp increase in the DC conductivity of PLA–CNT composites is observed. Then, $\sigma_{DC}$ clearly increases in the filler loading range (0.71≤φ≤1.07 vol.% CNT); the magnitude of $\sigma_{DC}$ at the upper limit is 7 times higher than the $\sigma_{DC}$ at 0.71 vol.% CNT. A sufficient number of conductive nanoparticles transform the composite from an insulator to a conductive due to the continuous linkage of filler particles, and a significant change in the conductivity of the composites is clearly seen at a critical point known as the percolation threshold $\varphi_{c}$ [42]. Accordingly, no significant change in $\sigma_{DC}$ occurs until the $\varphi_{c}$ of the filler is reached. At this critical point, $\varphi_{c}=0.71$ vol.% CNT, called the percolation threshold point, and the electrical resistivity of PLA–CNT dramatically decreases [43]. In fact, below the percolation threshold, the three-dimensional (3D) conductive network is not built from stem to stern [44]. Any further addition of nanofiller creates more networks and/or reduces the distance between nanofiller particles and significantly contributes to the rise of electrical conductivity [45]. The use of a scaling percolation law is a convenient framework to estimate the actual percolation threshold in hybrid materials, as in the case of polymer–CNT nanocomposites, where CNT is homogeneously or randomly distributed in the insulating matrix as stated in Ref. [45].

The fitting of the electrical DC conductivity relies on the plotting of $\log\sigma_{DC}$ versus $\log(\varphi -\varphi_{c})$, where the value of $\varphi_{c}$ was increased until achieving the best linear fit, given by the highest correlation factor. The fitting results showed that the percolation threshold is $\varphi_{c}=0.71$ vol.% (1 wt.% CNT) and the critical exponent is μ=4.5. The value of μ is higher than the theoretical value, which is close to 2.0. The critical exponent depends on the lattice dimensionality; it is around 1.3 for a two-dimensional system and about 2 for a 3D system, as reported in several studies [46]. The values of the *k* exponent ranged from 1–8, as stated in Ref. [46]. While numerical modeling showed smaller values (e.g., 1–3), the higher value of *k* is attributed to the high number of nanoparticle clusters that are created and statistically increase above the percolation threshold point as provided from the DC conductivity function volume fraction curve. One cluster promotes the emergence of other clusters in its vicinity. The dispersion of MWCNTs is therefore highly sensitive to the conditions during the melt-blending process; the additional MWCNT weight concentration during blending has to be well monitored in order to control the number of undispersed clusters [47]. The low values of the percolation threshold are related to the high aspect ratio of the filler; the different distribution of the CNTs in the polymer matrix (e.g., ordered distribution) can be obtained during melt processing, where polymeric chains are covered by CNTs on its surface [48]. The higher aspect ratio of the fillers leads to the lower value of the percolation threshold. On the other hand, the distribution of CNTs in the polymer matrix is not uniform. The ordered distribution of the filler is a result of the composite preparation procedure. The mixture of PLA and CNT powders creates a structure where big particles of PLA appear to be covered by carbon nanotubes, so that the conductive phase of CNTs is on the surface of the polymer particles. Hot pressing deforms the polymer particles and results in the formation of a compacted continuous polymer phase, where conductive patterns of filler are located on the boundaries between pressed polymer grains [49,50].

### 4.2. Prediction of the Percolation Threshold in RF Regime

The dielectric properties of PLA–CNT composites were measured in the microwave/RF regime corresponding here to the Ka band, i.e., the 26.5–40 GHz frequency range. According to Equation (2), RF conductivity $\sigma_{RF}$ is proportional to the imaginary part of permittivity and to frequency; as the frequency increases, the RF conductivity increases too. For lower concentration, the conductivity is increasing with frequency, attributed to the polarization currents of fixed dipoles [51].

**Figure 2:**
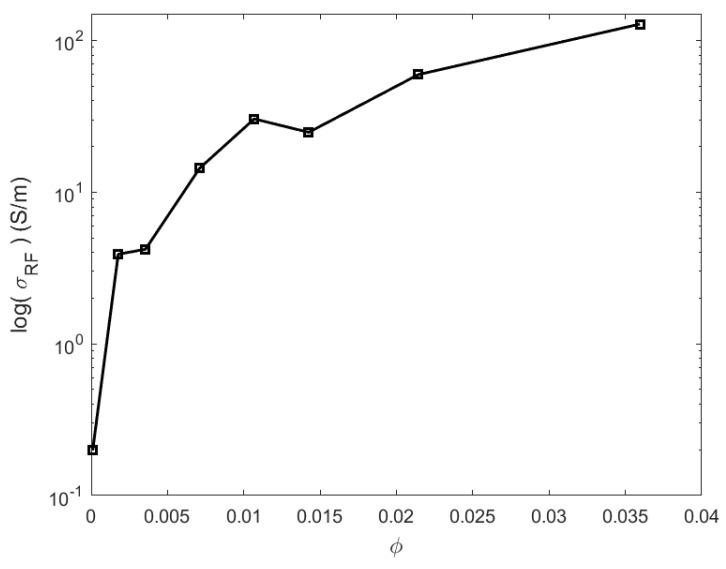
Logarithm of the microwave RF conductivity averaged over the Ka frequency range in function of CNT volume fraction.

The variations of RF conductivity against CNT content are presented in Figure 2. The logarithm of the RF conductivity averaged over the 26.5–40 GHz frequency range is plotted versus the CNT volume fraction. The pure PLA in the microwavre shows a conductivity of about 0.11 S/m. The literature reports values as low as 0.0164 for the loss tangent factor of pure PLA around 40 GHz; this corresponds to a conductivity of 0.12 S/m, close to the range observed in Figure 2, and confirms that neat PLA is not strictly insulative in the microwave regime [52]. As reported in Refs. [12,53], the electrical percolation is directly reached in the GHz range since the lowest charged composite (PLA–CNT at 0.25 wt.% CNT) exhibits an effective conductivity of about ∼3 S/m, which is drastically higher than pure polymer. There is an abrupt increase in the RF conductivity from the pure polymer up to a volume fraction of φ=0.01 (or 1.5 wt.% CNT). The rise of conductivity is obviously due to the short-circuiting of close CNTs workable at microwave frequencies. Above ∼1 vol.% CNT, the change in conductivity becomes marginal and almost maintains a plateau. This can be attributed to the effect of nanocharge agglomerates that reinforce the conductive network without enhancing the effective conductivity. The percolation threshold at microwave for PLA–CNT composites is concluded to be lower than our minimum CNT concentration (i.e., 0.25 wt.% CNT) mentioned earlier [39].

### 4.3. Prediction of the Percolation Threshold through the Mamunya Model

**Figure 3:**
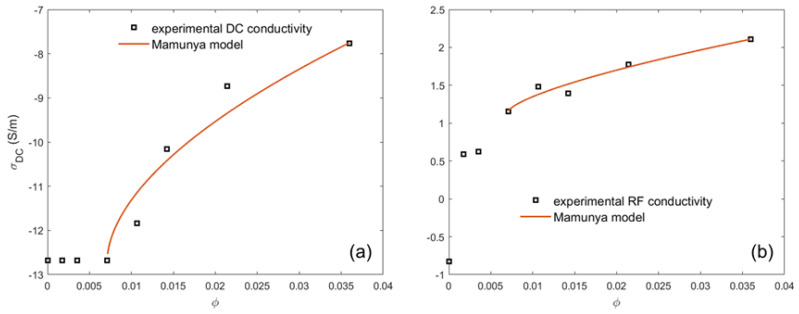
Theoretical and experimental plots of DC (**a**) and RF (**b**) conductivities against the volume fraction of CNTs based on Mamunya model for PLA–CNT conductive composite systems.

Based on experimental DC and RF conductivities, the percolation threshold and the maximum packing fraction are the two specific points that are required for the application of this model. Generally, the Mamunya model curve accurately predicts the electrical conductivity, specifically at the endpoint volume fractions, where the calculated and measured conductivity values are quite the same. The simulation process according to Mamunya can diverge from the measured DC and RF values for volume fractions in between the endpoints, as clearly seen in Figure 3. Below the percolation threshold, there is no accordance between the measured and the calculated values according to Mamunya. In our previous work [25]. Models based on the effective medium theory were used to describe the RF electrical conductivity of the CNT-based polycarbonate composites. The Mamunya model characterized with adjustable parameters was used; it showed good agreement between the experimental results and the calculated values. In different carbon nanotube composites, the percolation threshold is stated in Ref. [25] for different matrices and for another type of nanofillers as carbon black in Ref. [54].

### 4.4. Prediction of the Percolation Threshold through the McCullough Model

**Figure 4:**
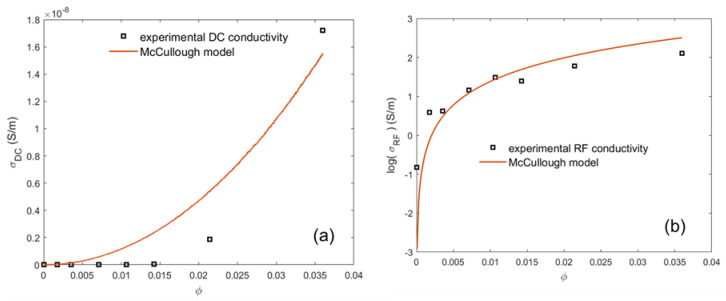
Theoretical and experimental plots of DC (**a**) and RF (**b**) electrical conductivity against the volume fraction of CNTs based on McCullough model for PLA–CNT conductive composite system.

From Figure 4, it appears that the McCullough model accurately follows the experimental DC conductivity data at/above the percolation threshold delimited by the filler volume-loading region (0.5–1.5 wt.% CNT), where an upsurge of conductivity is observed. The theoretical McCullough curve fits with measurement for λ=0.98. The filler shape factor λ=0.98 (which is maximum equal to one) indicates the formation of clusters of CNTs in this region. Furthermore, the applicability of the McCullough model is favored at 0.5≤φ≤1.5, where percolation appears. This observation can be explained by the tendency of CNTs to create long-chain linkage in this region. As the volume filler loading is increased, the McCullough model fails to predict the conductivity, which can be explained by the decrease in the RF conductivity for (PLA–2 wt.% CNT), and as we increase the filler loading the McCullough conductivity is above the experimental conductivity, which might be explained by the reduction in the extent of chain branching. In the case of DC conductivity, according to McCullough, the extent chain branching is λ=1; this model gives good results at $\varphi_{c}\leq 1$ wt.% CNT; as we increase the nanofiller content, the McCullough model for DC conductivity does not fit well as the theoretical values are above the experimental ones. The application of the McCullough model to predict DC and RF conductivities is favorable at only specific concentrations of the nanofiller. The higher value of the chain branching required to obtain a good fitting makes possible the hypothesis of chain breakage during the elaboration of the nanocomposites at a specific concentration of the filler as theoretical DC and RF conductivity diverges from the measured values; this speculation is independent of the percolation threshold point, which makes the assumption of a creation of a high CNTs network as the values of λ for both theoretical DC and RF conductivities are close to 1.

### 4.5. Prediction of the Percolation Threshold through the GEM McLachlan Model

**Figure 5:**
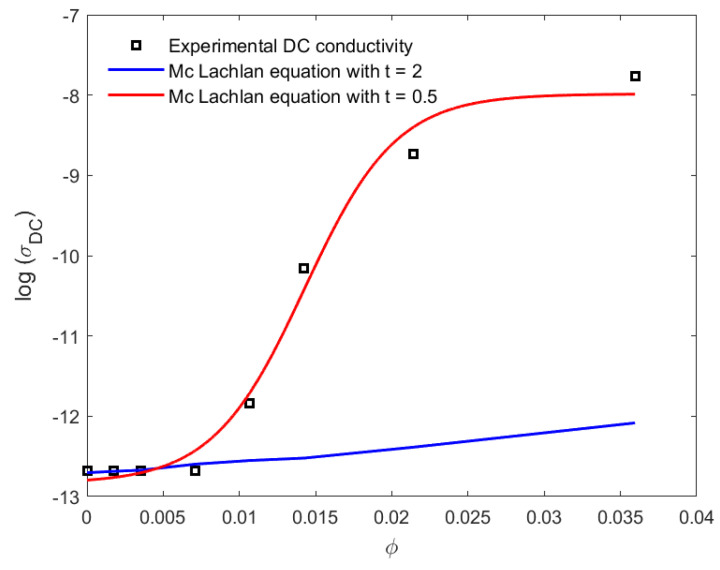
DC conductivity as a function of CNT volume fraction. The full squares are the measurement; the solid curve is a calculation based on the McLachlan equation, Equation (2), with t=2, and the dash-dotted curve is a calculation based on Equation (10) with t=0.5.

**Figure 6:**
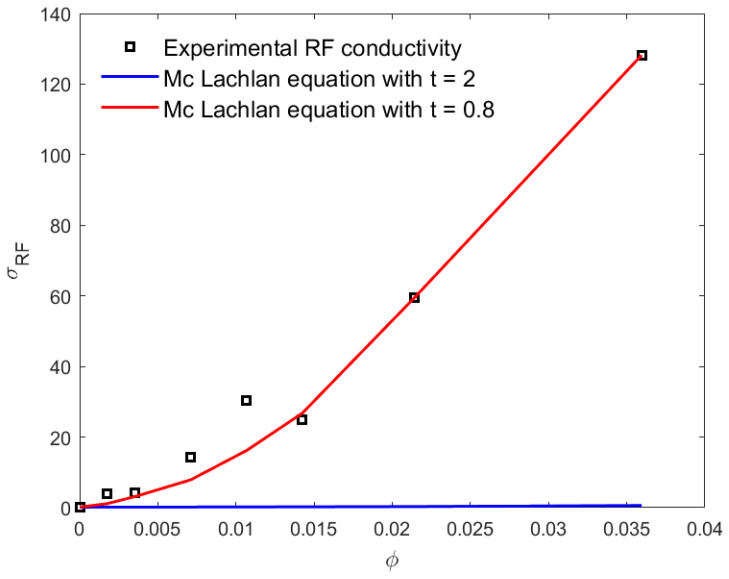
RF conductivity as a function of CNT volume fraction. The full squares are the measurement; the solid curve is a calculation based on the McLachlan equation, Equation (2), with t=2, and the dash-dotted curve is a calculation based on Equation (10) with t=0.8.

In Figure 5, we illustrate the simulation of the experimental electrical conductivity data for the PLA–CNT nanocomposites using the GEM McLachlan model. As seen from the analysis of Equation (10) (see Figure 5), for the composition below the percolation threshold $\varphi_{c}\leq 1$ wt.% CNT, the DC conductivity is predicted with t=2 as the critical index; as we increased the value of CNT weight concentration above the percolation threshold point, both DC and RF electrical conductivities as shown in Figure 4 were fitted as we decreased the value of t to, respectively, 0.5 and 0.8. These values are in accordance with those reported in the literature [24] and are close to the ones found in Section 4.3 according to Mamunya model. Figure 5 and Figure 6 show that the GEM equation of McLachlan is independent of/dependent on the percolation point for prediction of RF and DC conductivity, respectively. The lower values of *t* are also found in the literature [43,45]. The lower values of the exponent *t* than the universal values may be ascribed to a wide CNT distribution due to a large range of effective geometrical resistivity factors in a continuous homogeneous conducting phase [45].

### 4.6. Dielectric Constant and Dielectric Loss

The dielectric constant of a material is one of the fundamental features that is used to determine the electrostatic properties such as energy storage capability under the influence of an external electric field [55,56]. The charge screening effect is associated with the addition of MWCNTs to the PLA matrix on the dielectric permittivity response under the electromagnetic field. The polarization effects associated with the PLA and PLA–MWCNT interfaces are reviewed in this section.

**Figure 7:**
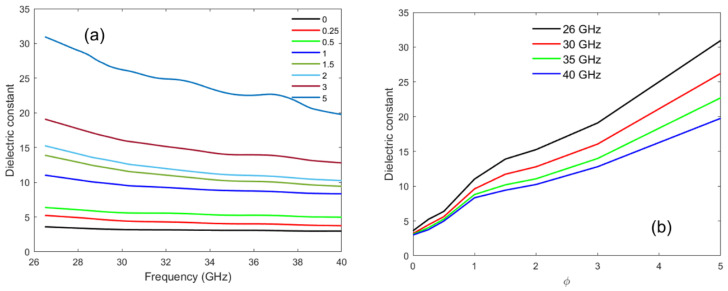
Dielectric constant as a function of frequency (**a**) and CNT weight fraction (**b**).

The dielectric constant $\varepsilon^{\prime }$ determines the ability of a material to store electrical energy under the influence of an external electric field. Figure 7 shows the dielectric constant of the PLA–CNT nanocomposites as a function of frequency (in a) and MWCNT concentration (in b). For the PLA nanocomposites with low MWCNT loadings (0.25 and 0.5 wt.%), there is a marginal decrease with increasing frequency. As MWCNT loading is increased to 1 wt.%, the PLA–MWCNT nanocomposites with 1 wt.% MWCNT concentration exhibited an average dielectric constant over the spectrum of 6 to 28. The most loaded composite (PLA–5% CNT) shows the average dielectric constant of 25.5. Compared to the literature, one sees those values are in agreement with well-dispersed CNT composites [45,53,57,58,59]. The loss tangent is significantly higher too because of higher imaginary permittivity [53,57,59]. The mechanical squeezing during hot-pressing is probably the cause of the high conductivity since the nanocharges are confined in a very thin film instead of three-dimensional bulk material [60,61,62].

The dependence on the frequency of the dielectric constant of PLA–MWCNT composites at different MWCNT concentrations can be modelled by a micro capacitor/resistor network. In PLA–MWCNT composites, PLA interspacing between MWCNTs forms a network of micro-capacitors of varying length scales. The dielectric constant depends on the number of micro-capacitors and the isolation distance between adjacent MWCNTs [63]; it considerably decreases by increasing the MWCNT concentration. Hence, the overall dielectric constant is remarkably enhanced since it depends on the micro capacitors that are inversely proportional to the distance. At high MWCNT concentration, the electron charges in CNT migrate and accumulate at the polymer–MWCNT interfaces, creating their polarization. Such polarization caused by trapped/accumulated charge carriers at the interface is called interfacial or the Maxwell–Wagner polarization effect [64]. It is responsible for the high dielectric constant at low frequency. But at high frequency with an applied electric field, the probability of these space charges to drift and accumulate at the interface is reduced; also, the capacitance between charges tends to short-circuit so that the dielectric constant of PLA–MWCNT nanocomposites decreases at high frequency and tends to that of neat PLA.

Percolation theory can once again be used to elucidate the dielectric properties of nanocomposites. A significant change in the dielectric constant is anticipated when the volume fraction of randomly dispersed conductive fillers approaches the percolation threshold. At this threshold point, numerous conducting phases are separated by insulating thin dielectric layers, rendering the system heterogeneous. Consequently, such nanocomposites in the proximity of the threshold point possess excellent charge storage capacity and can work as capacitors. The presence of an inhomogeneous electric field distribution in these materials further enhances their dielectric constant values. The dielectric constant values, as depicted in Figure 7, were fitted by using Equation (2), resulting in a derived percolation threshold value $\varphi_{c}=1.81$. This value aligns well with the universal range (1.6 to 2) observed in three-dimensional nanocomposites [65]. The theoretically calculated percolation threshold value closely corresponds to the experimental data. Typically, rod-like inclusions play a pivotal role in determining the percolation threshold as their intersection probability is higher than the spherical inclusions, thereby leading to a lower percolation threshold. Additionally, the substantial interface-to-volume ratio of nanotubes intensifies their interaction with PLA. Interfacial polarization, arising from space charge accumulation, including the Maxwell–Wagner–Sillars effect (MWS effect) and short-range dipole–dipole interactions (exchange coupling mechanism), may contribute to these phenomena [64].

**Figure 8:**
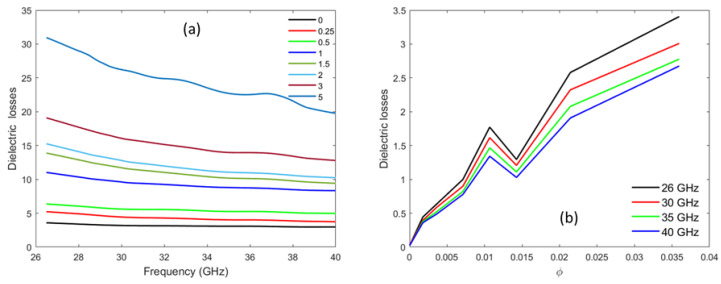
Dielectric losses as a function of frequency (**a**) and CNT volume fraction (**b**).

Dielectric losses result from the inability of charges to polarize at polymer interfaces when the frequency changes. Figure 8 represents the dielectric loss tangent factor, tanδ, of PLA–MWCNT composites versus frequency (in a) and versus MWCNT concentration (in b). The value of tanδ increases with the MWCNT concentration, ranging between 0.02 and 3.40 for the maximum concentration. The nanocomposites do not show much change in dielectric loss for an MWCNT concentration below $\varphi_{c}$. At the vicinity of the percolation threshold ($\varphi_{c}=1$ wt.%), dielectric losses undergo a visible change from 0.62 to 1. In this range of concentration, as the percolation occurs the CNT conductive network appears, which leads to significant conductivity. This affects the dielectric losses, which are proportional to the conductivity. A dip in dielectric loss is observed around 15 wt.% in Figure 8b. It can be related to the sudden increase in conductivity that occurs at the percolation threshold. Just above this threshold, it is possible that the charge dispersion locally reorganizes as conductivity decreases, affecting the dielectric loss proportional to it.

## 5. Conclusions

In this study, polymer composites based on carbon nanotubes as conductive filler were developed for conductive polymer composite applications. From the series of carried out experiments, it was found that the addition of CNT provides better in-plane conductivity. This indicates that the one-dimensional aspect ratio of the MWCNTs and their good dispersion into the PLA matrix provides better conductivity.

The conductivity predicted by the Mamunya model exhibits some similarity with the experimental DC and RF conductivities at a higher concentration of CNT at 1 wt.% and 0.25 wt.% respectively, as predicted as well from the percolation law. The prediction of DC and RF conductivities using the McCullough model is quite limited as the creation of the higher CNT network can be obtained at a specific concentration of CNT. The assumption of chain breakage may be presented as RF conductivity and be reduced at high CNT loading, as depicted from the microwave concentration. Meanwhile, the GEM model successfully predicted the through-plane electrical conductivity for all the produced composites. Through this study, the Mclachlan model is the standard model that better predicts DC and RF conductivities and shows the best agreement with the experimental results compared to the Mamunya, McCullough, and GEM models.

## Figures and Tables

**Table 1:** CNT weight and volume concentration in % with the corresponding name.

	PLA–0.25%CNT	PLA–0.5%CNT	PLA–1%CNT	PLA–1.5%CNT	PLA–2%CNT	PLA–3%CNT	PLA–5%CNT
**wt.%**	0.25	0.5	1	1.5	2	3	5
**vol.%**	0.18	0.36	0.71	1.07	1.42	2.14	3.59

## Data Availability

The data presented in this study are available upon reasonable request from the corresponding author.

## Abbreviations

The following abbreviations are used in this manuscript:

PLA	Poly(lactic acid)
CNT	Carbon nanotube
MWCNT	Multi-walled carbon nanotube
EMI	Electromagnetic interference
ESD	Electro-static discharge
CPC	Conductive polymer composite
RAM	Radar-absorbing material
DC	Direct current (i.e., static regime)
RF	Radio frequency (i.e., microwave regime)
GEM	Generalized effective medium
MWS	Maxwell–Wagner–Sillars effect
CCVD	Catalytic chemical vapor deposition
AR	Aspect ratio
